# Social Network Impacts and Moderators of Depression Among Indigenous Maya People Remaining in Place of Origin in the Migrant-Sending Guatemalan Western Highlands

**DOI:** 10.3390/ijerph22091328

**Published:** 2025-08-26

**Authors:** Haley M. Ciborowski, Kimberly C. Brouwer, Samantha Hurst, Ramona L. Perez, Kate Swanson, Holly Baker

**Affiliations:** 1 Herbert Wertheim School of Public Health and Longevity Science, University of California, 9500 Gilman Drive, La Jolla, San Diego, CA 92093, USA; kbrouwer@health.ucsd.edu (K.C.B.); shurst@health.ucsd.edu (S.H.); hshakya@health.ucsd.edu (H.B.); 2 Department of Geography, San Diego State University, 5500 Campanile Drive, San Diego, CA 92182, USA; 3 Department of Anthropology, San Diego State University, 5500 Campanile Drive, San Diego, CA 92182, USA; perez@sdsu.edu; 4 Department of International Development Studies, Dalhousie University, 6299 South St, Halifax, NS B3H 4R2, Canada; kate.swanson@dal.ca

**Keywords:** migration, depression, mental health, social networks, CES-D, Guatemala, indigenous, Central America

## Abstract

Remaining in the place of origin while family, friends, and neighbors emigrate can have adverse effects on psychological well-being. Specific important relationships absent from one’s social network can be especially impactful, while other relationships and network characteristics still available in the home network can be protective against psychological distress. The highlands of western Guatemala experience emigration at high rates and changing social network structures, affecting the mental health of those remaining at home. This study uses socio-centric network data from a single community (*N* = 653) to investigate the association between having emigrant ties in the United States and experiencing depressive symptoms according to an adapted CESD-20 scale. We also explore which types of relationships and network characteristics increase the likelihood of reporting depressive symptoms or moderate the relationship between emigration and depression. Our results indicate that having emigrant ties and more of them increases the odds of depression, even if only one friend or neighbor emigrated. Those with lower levels of education were also more likely to report depressive symptoms. However, more connected networks offered some protection from depression. Certain critical relationships still available at home, like a mother or sibling, lowered the likelihood of depression. For women, higher transitivity, or network cohesiveness, moderated the relationship between emigration and depression, and for men, a higher proportion of their connections outside of the household than within the household moderated that relationship. These findings may offer some insight into important relationships and network structures that may be leveraged to ease the mental health burden for those remaining at home while friends and loved ones emigrate.

## 1. Introduction

There is evidence that remaining in one’s country of origin as part of a migrant-sending community is associated with depressive symptoms [1,2,3,4,5,6,7]. Previous research suggests reduced psychological well-being and emotional health, and increased loneliness and anxiety in migrant-sending populations worldwide [8,9,10,11,12,13,14,15,16,17,18,19,20,21,22,23]. Studies utilizing depressive symptoms as a mental health outcome found evidence of significantly increased depressive symptoms in those remaining in their home country [14,18,23,24,25,26,27,28,29,30,31,32,33,34,35]. Unauthorized migration from rural Guatemala to the United States has become a normalized strategy for household economic advancement [36]. According to the United States Department of Homeland Security, there are roughly 750,000 unauthorized immigrants from Guatemala living in the United States. The effects of climate change are expected to increase migration pressure [37]. Rural areas in Guatemala are particularly vulnerable, as changing precipitation patterns and extreme weather events reduce the economic viability of agriculture [38]. High volume out-migration drastically changes social network structures and shifts cultural norms for communities in countries of origin [39]. These changes can dramatically influence mental health for those remaining in their home communities while important network ties migrate away [40]; however, there is a lack of migration research specifically using social network analysis techniques to explore how social network characteristics may impact and moderate mental health outcomes. In this study, we use socio-centric network data from a representative community of Maya Peoples in the rural western highlands of San Marcos, Guatemala, to explore the effect of emigration on depressive symptoms and the social network characteristics that may moderate that relationship. Our primary research questions were (1) does having close friends and family ties emigrated to the US impact the odds of reporting depressive symptoms, and (2) what network characteristics may moderate that association. We hypothesized that having close ties to those emigrating to the US, and more of them, will increase the odds of depressive symptoms. The network relationships that may moderate or buffer that association are an exploratory question, though we anticipated there would be relationships that act as effective moderators. This is the first known study to utilize social network data to explore the relationship between migration, social network structure, and mental health in this region.

There are mental health consequences of navigating the transnational familial structures formed in communities with normalized migration. Transnational families represent global care chains, where there is understood mutual sacrifice at home and abroad for the greater good of the household [41]; however, these structures also represent a rupture in traditional familial structures where both mother and father, grandparents, and siblings are residing within the home or community [42]. These separations are navigated through telephone and internet communication, and financial remittances that afford better homes and educational opportunities for children, but they are also spaces of loss, longing, mistrust, and lack of direct parental care [42].

Studies with women remaining in their home countries while spouses and other network members emigrate domestically and internationally reveal varying experiences and consequences [17,28,30,43]. For example, studies with Mexican women with emigrated spouses, children, or other close ties experience elevated levels of mental health distress [17,43,44,45]. However, it is important to acknowledge that the impacts of migration are not implicitly negative on women remaining in their place of origin. There are often positive changes in traditional gender roles, resulting in more independence for women. A study with Maya in the Yucatan by Bever (2002) argues for the benefits of transforming gender roles through migration, bringing more autonomy and control of household resources for women [46]. A study by Taylor, Moran-Taylor, and Ruiz (2006) with four rural communities in Guatemala also reported female empowerment through migration. They provide accounts of female autonomy, escape from oppression and abuse, and increased participation of women in emigration [47]. However, both studies reported the short-lived nature of changes in gender roles, attributable to deep cultural norms and social structures resistant to change [46,47]. Men remaining at home in migrant-sending communities are also impacted. Witnessing the ability of other households to provide educational and economic opportunities for children and families can lead to feelings of inadequacy and failure for men unable to migrate successfully [42,48]. Men who have migrated previously and been deported or have had unsuccessful migration attempts may be at particular risk of mental health struggles. Unauthorized migration attempts from Guatemala to the United States can cost more than a yearly household income, even with someone sending remittances from the US. Failed attempts result in overwhelming debt, the loss of land and homes used as collateral, and often result in multiple migration attempts to alleviate that debt [49].

Physical, political, and structural threats of violence may also contribute to poor mental health for Indigenous populations in the highlands of San Marcos, Guatemala. Members of these communities fled to the rural highlands to escape genocide during the civil war and are still living with the trauma from that experience, while navigating new forms of structural violence. Neoliberal policies diminished economic opportunity for Indigenous Guatemalans and created space for violent criminal activity in border regions [50]. Gang activity is prevalent in the district of San Marcos, and this area is home to the largest operations of poppy cultivation in the country [51]. Indigenous Guatemalans already living with the memory of genocide in their communities during the civil war are now living under constant threat and fear of gang violence. These forms of violence can compound mental health stressors when intersected with structural inequities like diminishing land availability, climate change impacting crop yields, environmental health dangers from industrial mining operations, lack of economic and educational opportunities, and the militarization of borders threatening migration as a survival strategy [38,52,53,54,55,56,57,58].

### 1.1. Social Network Effects

Emigration causes changes in the social network structures of migrant-sending communities [39,59]. Studies examining the association of poor mental health and remaining in place of origin often identify the vital role of social ties against poor mental health outcomes [20,27,29,60]. For instance, some research has found that social ties and network cohesion moderate the association between migration and poor mental health [7,10,11,27,29,45,61]. Migration research has rarely employed social network analysis to examine how network characteristics might influence mental health outcomes. The moderating effects of relationship types and network size described in social support literature suggest an opportunity to investigate the moderating effects of social network analysis variables in the relationship between migration and poor mental health.

Network data can be either ego-centric or socio-centric. Ego-centric network data focuses on the personal network characteristics of an individual (also known as an “ego” in network terms) [62]. Socio-centric network studies map all connections within a small, bounded network [63,64]. Measures that may provide insight into mental health outcomes include those that describe structural position within a network and measures of network size [65]. Transitivity is a measure of structural position that reflects network cohesion by quantifying the probability that an individual’s network connections are also connected to one another [66,67,68,69,70]; or more simply, the extent to which an individual’s friends are also friends with one another. Network size is most commonly measured by degree, or the total number of connections an individual has within a network [62]. The in-degree describes the number of people that have nominated an individual as a part of their network, while the out-degree is the number of other people (also known as alters in network terms) that an individual names [62]. Research suggests that individuals with smaller networks are more likely to experience depression [71]. Research with adolescents provides evidence that social isolation, measured as total degree, and low transitivity are associated with poor mental health outcomes and suicidal ideation [72]. Depression outcomes are also influenced by network centrality, or how connected an individual is to others in the network. For example, in a longitudinal study with more than 12,000 participants, Rosenquist, Fowler, and Christakis (2011) found that higher network centrality scores significantly decreased the likelihood of depression [65].

There is evidence of sex differences in the association between network measures and depressive symptoms [73]. Feminist migration and geopolitical literature identify the gendered costs of migration-based changes to a woman’s network. For example, a study by Torres and Carte (2016) on migrant-sending populations in rural Mexico found that migration places demands on women to become heads of household, part of the labor force, and managers of financial remittances in addition to their traditional roles and caring for children, the elderly, and the infirm [74]. They also note that women and older adults endure heavy workloads and care for children alone, causing children to suffer when over-extended caregivers cannot provide adequate supervision and support; these changes result in psychological and emotional stress, depression, and substance use, and perpetuate migration [74].

### 1.2. Mental Health in Guatemala

Information on mental health in Guatemala is scarce. While there is a mental health plan in place in Guatemala, no funding is allocated to implement the plan, and no official mental health expenditure was reported on the 2020 World Health Organization mental health report [75]. According to that same report, there were just eight studies published on mental health in Guatemala since 2019. Naber (2022) used the 2016 National Disability Survey to examine characteristics of people with anxiety or depression in Guatemala. They report higher odds for women, older adults, urban dwellers, and those who are divorced, separated, or widowed [76]. Most existing mental health studies in Guatemala concern experiences of violence during and following the civil war. Sabin (2006) performed a study of 170 Guatemalan refugees aged 16 and older living in Chiapas, Mexico. Post-traumatic stress disorder, anxiety, and depression were common within that population, including 47.8% of 179 participants meeting symptom criteria for depression [77]. Keller (2017) surveyed 234 adults who had migrated from the Northern Triangle, 87% of whom reported direct exposure to violence or threats. Most other participants reported violence in their communities as the reason for immigration; twenty-four percent of that sample met the diagnostic criteria for depression [78]. Puac-Polanco (2015) surveyed 1452 adults who had experienced violence during and following the civil war in Guatemala. Of that sample, 4.2% screened positive for depression. Women, Indigenous Maya, and urban dwellers had greater odds of experiencing poor mental health [79]. Branas (2013) found associations between violence and mental health, with 40.7% screening positive for depression among 86 people from Guatemala City and the Sololá department in the western highlands. Those rates were higher than expected but attributed to ongoing violence and fear since the end of the civil war because 90% of participants reported still fearing violence [80]. Sabin (2006) surveyed 179 previous refugees who had returned to Guatemala after living in refugee camps in Mexico and found that 47.8% met the symptom criteria for depression [77]. No other studies were focused on depression incidence in Guatemalan populations outside of experiences of violence, highlighting the need for future research in this area of study. In general, it may be difficult to study and quantify the mental health struggles of Indigenous Guatemalans using Western mental health measures. The way these emotions are experienced is unique to the cultural realities and lived experiences of these groups. For example, in Latin America, the concept of *nervios* is widely recognized as a depression-like disorder, often affecting women and children experiencing loss due to migration. *Nervios* is marked by melancholy, extreme sadness, anger, and even violence stemming from loss and heartache [81]. Grandmothers and wives remaining at home caring for children and the elderly may experience *tristeza*, a general sadness that lives under the surface, but becomes another part of daily life [52]. For those who lived through the civil war, the experience of painful emotions may not manifest in ways that can be measured in Western academic terms, as many carry with them a general trauma and sense of guilt that also becomes a normalized part of life [52].

### 1.3. Gendered Effects

While Guatemalan Indigenous women are immigrating north more often, they are still the dominant group remaining at home while spouses, older children, and their parents emigrate [47,82,83]. Historically, Indigenous women in Guatemala have faced mental health consequences from losing loved ones to violence, political persecution, and emigration, including during the 36-year civil war that ended in 1996, in which thousands of Indigenous Maya were victims of genocide [84,85]. Research on Indigenous women in Guatemala has documented mental health distress from the direct loss of community, fear of violence, and economic insecurity [83,86,87,88]. For example, Sanchez Ares and Lykes (2016) documented feelings of suffering and abandonment among Southern Quiche women in Guatemala who had been left behind by emigration [83]. Women in that study also expressed experiencing pressure to migrate for economic survival and feelings of responsibility to care for other women and girls left behind by emigration [83]. On the contrary, there is also evidence of female empowerment and autonomy among left-behind women in Guatemala. Women may benefit from independence through escape from oppression or abuse and gain confidence in household decision-making [47].

### 1.4. Historical Context

Within this study population, network ties may be complex, as communities in this region are closely connected through familial relations but have a complicated and traumatic history. During the 1980s, throughout the civil war in Guatemala, thousands of Maya were victims of Indigenous genocide [85]. According to the Comisión para el Esclarecimiento Histórico (CEH), the government regime targeted civilians to limit rebel support, and 83% of victims were Indigenous Maya [89]. The CEH estimates 200,000 people were executed, another 200,000 became refugees, and one million were internally displaced [89]. Most of the massacres were carried out by civilian patrols, often made up of members of one’s own ethnic group [90,91]. According to both recorded truth commission accounts and local oral history, this study population experienced this government-sanctioned Indigenous genocide, including individuals forced to kill others in their own communities. The experience has led to mistrust even between families still living as neighbors.

### 1.5. Theoretical Basis

While the history of this population may be complicated, social network data may reveal important features of a rural community experiencing large-scale out-migration. This exploratory study drew from the buffering hypothesis in social support literature. The buffering hypothesis is the theory that social relationships can act as a buffer against depression, anxiety, and other mental illnesses following a traumatic experience [92]. In line with the buffering hypothesis, we investigated whether social network characteristics buffer against negative mental health impacts following the experience of network ties migrating to the United States. Previous research suggests that even the number of social ties may mitigate the adverse mental health effects of remaining in place of origin to migration [27,45]; however, it is unclear how other characteristics of social networks may lower the odds of poor mental health among Indigenous Maya Peoples, even in the presence of missing family members. For example, centrality in one’s social network (being more connected or “popular,” depending on the centrality measure) may serve to buffer against depression through reducing isolation, increasing emotional and resource support, and a sense of belonging [93]. Conversely, network centrality may increase stress for connected individuals because of burdensome relationship management, influence effects, or behavioral spread, highlighting the importance of relationship quality over quantity [65,94]. To these ends, our research aims were (1) to first assess whether the migration status of close family and friends is associated with depressive symptoms in adults over the age of 15 in a rural Indigenous Maya community, and (2) then explore network characteristics that possibly moderate or “buffer” the relationship between having network out-migrants and symptoms of depression.

## 2. Materials and Methods

### 2.1. Study Population

We conducted a cross-sectional census survey with all residents of a single rural village in San Marcos, Guatemala (*N* = 653). Recruitment took place from 4 August 2018 to 30 June 2019. We defined the study population as men and women aged 15 years and older; links to others living outside this community were excluded. Due to the nature of this study site, subjects were all of Indigenous Maya ethnicity. Most participants could speak Spanish, though some older adults still speak Mam. Surveys were conducted in Spanish by the first author or a trained local health worker, and a Mam-speaking local guide was present for all interviews if translation was necessary. This project underwent a rigorous human subjects ethics review at the University of California, San Diego (IRB#181152S), especially because of the involvement of individuals under the age of 18. Within the communities of the study site, young people are often considered adults by the age of 15 and were included as essential viewpoints in this research. Informed consent was obtained orally and recorded on participant survey files because of the varied literacy rate in the community. Where minor study participants lived in households with parents present, oral informed consent was obtained from a parent. In some cases, minors were heads of their own households, which is culturally accepted in this Indigenous community, so they provided their own oral consent to participate. For this reason, we obtained a waiver of parental consent from the human subjects ethics review committee. Additionally, this study was reviewed and approved, and written consent was obtained from several official entities in Guatemala, including the Directors of the National Hospital in the study region, the Director of the Ministry of Health of San Marcos, and the Alcalde Auxiliar (local leadership) of this community. The safety of participants was considered at every point in the research process. The first author has been working alongside these communities for almost two decades and has built a trusted relationship. Without this confidence, the study would not have been possible. Due to the delicate nature of the study topics (unauthorized migration and depression), there is always the possibility of bias in responses, but this relationship gives us some assurance that response bias was minimal.

Participant households were from a single village in order to use social network analysis techniques to map the interconnected networks of the entire village. The village was chosen non-randomly based on the population size, the possible number of respondents meeting the inclusion criteria, and geographic centrality within the district; this choice was also made under the advisement of local leaders. The typical village population in this health district is roughly 500–1500 residents. While some communities have fewer emigrant ties, economic resources, and more modest homes, others have residents with more economic mobility, primarily due to remittances from the United States. Community leaders chose the study village as it was considered representative of surrounding villages in terms of socio-economic status, migration, and substance use. Households within the community were made aware of the study in advance by local leadership. The response rate was 99.8%, with one resident refusing participation.

### 2.2. Exposure and Outcome Variables

For network analysis, we used name generator questions to record significant network ties for each participant. Name generator questions are used within social network survey instruments to elicit network ties for each individual [95] Name generator questions included the following: whom they trust to confide in about something personal or private; whom they pass their free time with; whom they consider their closest friends; and how many friends or family members they see more than once a month that live outside of the community. We also asked questions to elicit the names of their spouse, parents, and siblings. The constructed edge list from the name generator questions was *N* = 5190. The range of named alters was 1–27, and μ = 8. The range of total network ties for an individual (total degree) was 1–45, and μ = 13.

The primary outcome variable was a binary threshold score of depressive symptoms, measured using the Spanish translation of the Center for Epidemiologic Studies-Depression scale (CES-D) [96]. The CES-D is a 20-item scale that records responses about feelings and behaviors over the past week. Scores are summed with a range of 0–60, with higher scores indicating more depressive symptoms. Numerous studies have validated this scale, including among Spanish-speaking populations in Latin America. Most studies testing the internal consistency of the Spanish version of the measure report a Cronbach’s α > 0.90. It is considered the most effective way to quickly measure depressive symptoms in a survey setting when more extended psychological interviews with mental health professionals are unavailable [96,97,98]. Questions in this scale were tested with community members from each age set and gender for language appropriateness, and adjusted as necessary. For example, the concepts of *nervios* and *tristeza* were sometimes used to describe what is meant by depression, stress, or anxiety in the questions where there was no cultural or language equivalent for participants. These concepts are more widely understood and accepted within this population as resembling what Westernized words describe as feelings of anxiety and depression. The standard threshold for risk of major depression using this instrument is 16, which we used to create the binary outcome for the presence of depressive symptoms [99,100,101].

Within this study, we were not able to perform clinical interviews and diagnoses from psychiatric professionals against which to validate the use of the CES-D scale with this population; however, previous studies with Indigenous, refugee, migrant, and other populations remaining in place of origin can provide us some theoretical basis to justify the use of this tool in the absence of clinical diagnoses. The validity of the CES-D questionnaire has been tested among other Indigenous populations, including Latin American Indigenous [102,103,104,105]. Some of those studies support the measure’s validity; others note cultural problems with the tool while still acknowledging its value in the absence of more intensive diagnostic measures [106,107,108,109,110,111,112,113]. Within this study sample, the scale demonstrated excellent internal consistency (Cronbach’s α = 0.91, 95% CI [0.90, 0.92], giving us a broad sense of depressive symptoms within this population. As these data are a census of the entire population of a single village, we can make some general observations about participant outcomes within the village, even if they cannot be generalized beyond this study population [114].

We asked participants whether they have any close friends or relatives who migrated away to the United States as a dichotomous “yes” or “no” response. They were also asked to report the number of their friends and relatives who migrated to the United States. Finally, we asked about the nature of the relationship with those ties. Those relationships included “spouse”; “parent”; “child”; “sibling”; and other relationships that fell under the umbrella of “close friend/neighbor,” which we collapsed into a single category.

Although it is beyond the scope of this paper, we asked participants questions from the *HCHS*/*SOL* alcohol consumption scale [115]. *HCHS*/*SOL* is the largest study that examined alcohol use and contributing factors among diverse Hispanic/Latino heritage groups. To create the alcohol exposure variable used in these analyses, we asked participants if someone in their household regularly drinks to intoxication. We included the alcohol exposure variable because household family members with alcohol problems may increase the risk and prevalence of psychological conditions and social trauma [116]. Living with an alcoholic spouse or partner can result in emotional problems and other psychological and social problems [117]. Having an alcoholic parent increases the likelihood of alcoholism and depression in their adult children [118]. The self-alcohol use variables were not included because of known social desirability bias in responses to self-alcohol use questions. Under-reporting was widespread, as family members of participants regularly stated that other participants living in their household regularly drink to intoxication, but those individuals denied their own alcohol use. Alcohol use most certainly influences and is influenced by depressive symptoms, adverse life events, and stressors; however, the complexity of this relationship warrants more attention than can be addressed in this current manuscript. These variable findings are reported here because they are worth noting and acknowledged as inextricably linked to depressive outcomes.

To test the possible moderating effects of certain social network characteristics, we used responses from the name generator questions to measure each ego’s network size and connectivity. Measures of network size included total “degree,” or the total number of network connections of an individual ego. Also measured were the “in-degree,” or the number of alters that nominated an individual, and the “out-degree,” or the number of alters named by an individual. As a measure of individual network cohesion, we used transitivity, or the probability that two of an individual’s network connections are also connected to each other [67,68,70,119].

Individual-level covariates included sex, age, marital status, level of education, religious affiliation, and perceived income sufficiency. Income sufficiency was tested as a collapsed dichotomous measure of income sufficiency adapted from the question “in general, would you say your income is…” with the following response options: “sufficient to live and save,” “sufficient to live but cannot save,” “not sufficient and there are some difficulties,” and “sufficient, and there are great difficulties.”

### 2.3. Analysis

We first characterized the association between falling above the CES-D threshold score of 16 for possible major depression and two versions of the exposure variable. The first was a dichotomized representation of whether a participant had even a single social tie that had emigrated to the US. The second was the continuous number of social ties a participant had while living in the US. We tested both iterations of the exposure variables for emigrant ties throughout this analysis. Only those that showed significance at *p* ≤ 0.05 are presented here.

After establishing the association between having any emigrant ties and higher numbers of emigrant ties, we investigated the demographic characteristics associated with depression. We built multivariate regression models to test the association between the emigrant tie exposure variables and depression in the presence of demographic covariates. We then tested the association of social network variables and falling above the CES-D threshold for possible major depression. We began with the types of relationships participants had with their emigrant ties in the US, followed by key relationships participants still had in their network at home, and finally, the calculated social network variables described below. We then tested the social network variables that were significantly associated with depression at *p* ≤ 0.05 as the exposure variables, replacing the two iterations of the emigrant tie variables in multivariate models. Finally, we tested the significant social network variables in multivariate models as interaction terms to investigate their possible moderating effects on emigrant ties and depression. We stratified significant interaction terms at multiple levels of the moderating variable for intuitive interpretation of the moderating effect.

To carry out these analyses, we first constructed a secondary dyadic dataset from the original dataset linking every ego with each of their named alters to map the network. Using that dataset, we then calculated the network size (degree) and cohesion (transitivity) measures. We then entered the calculated social network variables as additional variables for each individual in the original dataset. We conducted bivariate analyses to test for associations with falling above the CES-D threshold for depression (main outcome) using logistic regression and *X*^2^ tests. Those with a significance of *p* ≤ 0.10 were included in multivariate models along with covariates. We ran Generalized Linear Regression (GLM) multivariate models for the binary exposure of having emigrant ties in the US, and the continuous exposure of the number of ties emigrated to the US. We ran all models adjusting for demographic characteristics, income sufficiency, and total degree. Because the out-degree is included in the measure of total degree, we tested for collinearity among variables in the adjusted model. In the collinearity matrix, the out-degree and total degree shared a correlation coefficient of 0.79. We also calculated the variance inflation factor (VIF) of the adjusted model variables, with scores of 3.24 (out-degree) and 3.35 (total degree); while the VIF scores for those variables did not indicate severe multicollinearity in the model, the correlation coefficient was sufficiently high enough to err conservatively for concern about collinearity with a VIF > 2.5 [120]. Considering the population size of 653, we made the choice to omit the adjustment for total degree in models testing the out-degree.

For the significant network variables at the *p* ≤ 0.05 level in multivariate models, we constructed interaction models to test for moderating effects on the relationship between emigrant ties and depressive symptoms. For interaction models where the interaction term was significant at *p* ≤ 0.05, we then stratified the variables being tested as moderators to investigate the levels driving the moderating effect. Because previous research provides evidence of differences by sex for migration behavior and mental health outcomes [121,122], we also stratified all analyses by sex. The regression models presented in tables provide beta coefficients as parameter estimates, while the results in the text are presented as odds ratios.

## 3. Results

### 3.1. Aim 1: Emigrant Ties and Depressive Symptoms

Summary statistics are presented in Table 1. The proportion of participants with a CES-D score of 16 and above, indicating the presence of depressive symptoms, was 33% among all participants but 59% among those with one or more emigrant ties living in the United States. More than half of the study population had at least one emigrant tie in the US. Among women, 45% reported CES-D scores above 16, compared to 16% of men. Thirty-seven percent of participants reported a person who regularly uses alcohol to intoxication in their household. Of those, 39% had a CES-D score of 16 and above, as opposed to 29% for those without an alcohol-dependent person in the home.

In bivariate analyses (Table 2), having more emigrant ties was significantly associated with higher CES-D scores (OR = 1.10; 95% CI = 1.04–1.18). For each tie that had emigrated to the US, there was a 10% increase in the likelihood of falling above the cutoff for possible depression. Income sufficiency was not significantly associated with depression but was included as a covariate and proxy for income.

Looking at the relationships with those who have emigrated, having a close friend or neighbor who has emigrated was predictive of depression (OR = 1.53; 95% CI = 1.25–2.66). In terms of relationships in the home network, those with a mother or sibling in their home network were less likely to experience depressive symptoms. For the calculated network variables, a higher total degree and out-degree were associated with depression (OR = 1.03; 95% CI = 1.01–1.06). Higher individual network connectivity (transitivity) was associated with lower odds of depression in this population (OR = 0.11; 95% CI = 0.02–0.58).

We tested the binary exposure for having an emigrant tie and the number of emigrant ties in adjusted models for all participants. Having an emigrant tie was significantly associated with depression when adjusted for covariates (Table 3). In model 2, we added transitivity, which was significant for the reduced likelihood of depressive symptoms (AOR = 0.14; 95% CI = 0.02–0.89). Because the out-degree was also significant in the bivariate analysis, in model 3, we added the out-degree. As with the other two models with binary exposure for having at least one emigrant tie, sex, primary school, and household alcohol dependence remained significant. The additional network variable, the out-degree, was also significant and associated with an increased likelihood of depressive symptoms (AOR = 1.05; 95% CI = 1.00–1.09).

Models with the number of emigrant ties as the main exposure yielded similar results as the bivariate models (Table 4). As with the binary exposure, the number of ties was significant in all three models, indicating that as the number of emigrant ties increased, so did the likelihood of depressive symptoms. The addition of transitivity and the out-degree in models 2 and 3 each approached significance at *p* < 0.06. We eliminated other network variables that were significant in bivariate analyses because they failed to remain significant in the models.

### 3.2. Aim 2: Moderation Effects

**Figure 1:**
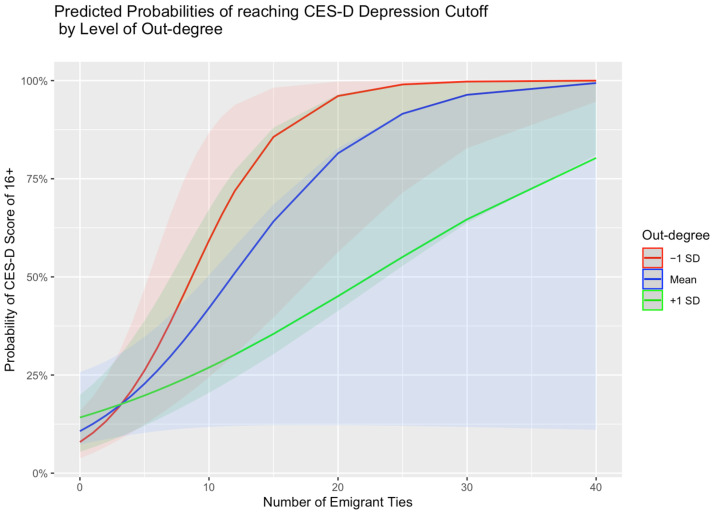
The predicted probabilities of falling above the threshold for symptoms of major depression associated with the number of emigrant ties at the mean level and +/−1 standard deviation of the out-degree, with 95% confidence intervals.

**Figure 2:**
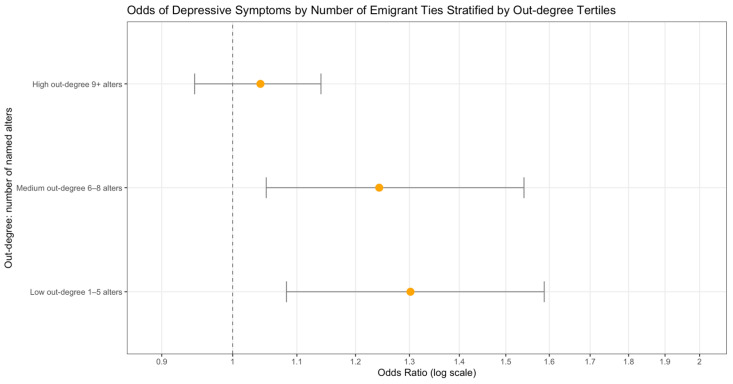
The odds of falling above the threshold for major depressive symptoms associated with the number of emigrant ties, stratified by three levels of the out-degree, with 95% confidence intervals.

The out-degree was a significant moderator in the relationship between the number of emigrant ties and depression, adjusting for all other variables from the models in Table 3 and Table 4. For the out-degree interaction model, the coefficient was negative, suggesting the effect of the number of emigrant ties on depression will decrease as the out-degree becomes larger (Beta = −0.023; SE = 0.01; *p* = 0.019). Figure 1 shows the predicted probabilities of falling above the CES-D cutoff of 16 for possible depression by the number of emigrant ties in the US. Overall, the trend shows that more emigrant ties increased the probability of meeting the threshold for depression; however, a lower out-degree increased the probability of depression even with a smaller number of emigrants in the US. With stratification at the median of the out-degree, those with a lower out-degree have a 1.32 (95% CI = 1.10–1.60) times higher odds of experiencing depressive symptoms based on their number of emigrant ties, compared to individuals with a higher out-degree, who have a 1.08 (95% CI = 1.02–1.17) times higher odds of depression based on their number of emigrated ties in the US. Participants with more emigrant ties are still more likely to be depressed than those with fewer or none, but the odds of depression are lower for those in the high out-degree subgroup, even with more emigrant ties. To investigate further, we stratified by tertiles, showing decreased odds of depression for each group as the number of named alters increased (Figure 2). We then explored what could be driving this unusual finding by calculating the proportion of out-degree nominations inside versus outside of each individual’s household, for both men and women. Men in the sample seem to be driving the significance of the out-degree. Men with a proportion higher than the mean of nominees in their household had higher odds of depression, while men with a higher proportion of out-degree nominees outside of the household had lower odds of depression. The household nominee proportion was a significant moderator in the relationship between the number of emigrant ties and depression, adjusting for all other variables from the models. The coefficient was positive in the interaction model, suggesting the effect of the number of emigrant ties on depression will increase as the proportion of nominees from within the household increases (Beta = 0.707; SE = 0.32; *p* = 0.026). The same effect was not detected for women in the sample.

### 3.3. Differences by Sex

In bivariate analyses for men, simply having an emigrant tie but not the number of ties was associated with depression (Table 5). Having a friend or neighbor who emigrated was significant among males. For relationships in the home network, having your spouse at home was associated with lower odds of depression, but having your father in the home network was associated with higher odds of depression. For network variables, those with a higher out-degree of named alters were more likely to be depressed. In adjusted multivariate models, only emigrant ties as a binary exposure were significant for males, with none of the tested moderators reaching significance (AOR = 2.65; 95% CI = 1.25–6.04).

Higher numbers of emigrant ties were associated with depression among women (Table 6). Having a friend or neighbor emigrate was associated with depressive symptoms, but having a mother or father at home in the network was associated with decreased odds of depression. For network variables, a higher total degree was also associated with depressive symptoms, and transitivity was significant for decreasing the odds of depression. The association between having more emigrant ties and CES-D scores of 16 and higher remained significant in multivariate models for women with all covariates (Table 7). There was an 11% increase in the likelihood of depression with each additional person who emigrated. For network variables, transitivity remained significant in the second multivariate model, as did the out-degree in the final model.

**Figure 3:**
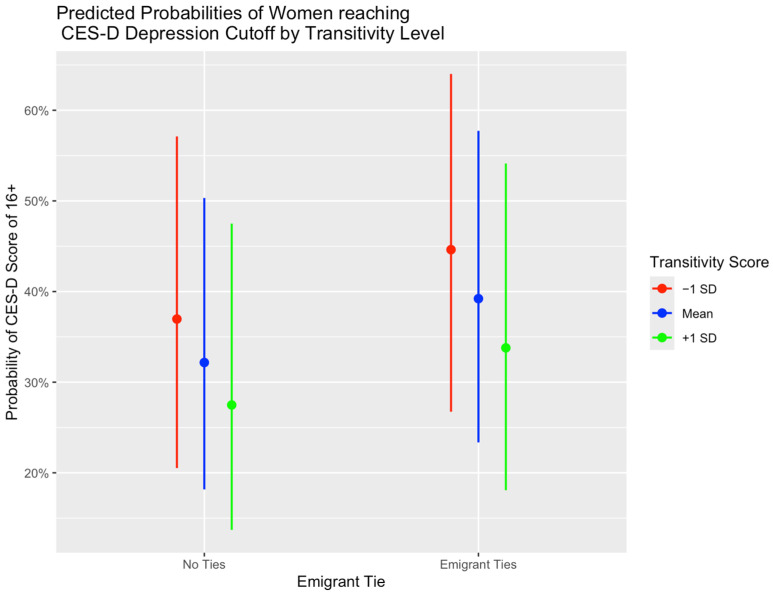
The predicted probabilities of falling above the threshold for symptoms of major depression for females with and without emigrant ties at the mean and +/−1 standard deviation of the transitivity score, with 95% confidence intervals.

**Figure 4:**
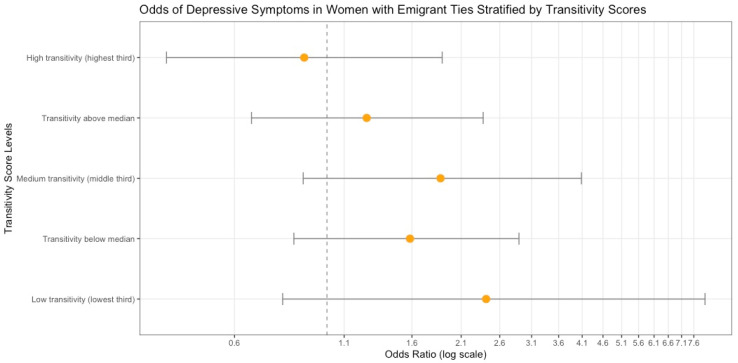
The odds of falling above the threshold for major depressive symptoms for females with emigrant ties (compared to those without emigrant ties), stratified by five levels of the transitivity score, with 95% confidence intervals.

To test for possible moderating effects of network variables among women, we ran models including interaction terms for those two variables. The out-degree was not significant in interaction models, but transitivity was a significant moderator between having emigrant ties and depression (Beta = −5.09; SE = 2.65; *p* = 0.05). The interaction coefficient was negative, suggesting that the effect of having emigrant ties on depression will decrease as transitivity becomes larger. For those with emigrant ties, having a more cohesive network resulted in a lower probability of reaching the CES-D threshold for depression; those without emigrant ties were less likely to report depressive symptoms, but higher transitivity still resulted in a lower probability of reaching that threshold (Figure 3). When stratified at the transitivity median, women WITH emigrants in the US and low transitivity or a less connected and dense network had a 1.54 (95% CI = 0.83–2.89) higher odds of experiencing depressive symptoms than their peers without emigrant ties. However, those who have more connected networks, while still more likely to be depressed than their peers without emigrant ties, only had a 1.25 (95% CI = 0.66–2.37) times higher odds of experiencing depression. When further stratified at transitivity tertiles, the differences in odds between groups can be seen with slightly more granularity (Figure 4). Estimates in the stratified analyses may not be precise, as confidence intervals are widened due to smaller sample sizes.

## 4. Discussion

### 4.1. Depression in the Rural Highlands

Communities in this region send individuals to the United States at high rates, as indicated by more than half of the study population having at least one and up to forty connections living in the United States. Immigration from this area has become a normalized survival strategy for economic growth and educational opportunity, creating an established culture of migration in the region [123,124,125]. Though remittances contribute to household well-being, they leave a gap in emotional capital that may not take the place of close relationships sacrificed in the process. Indeed, there are familial and friendship ties that are permanently ruptured and now navigated through transnational channels [42]. Though data on the overall prevalence of depression in Guatemala is scarce, a recent study estimated rates between 4 and 6% in those exposed to violence since the civil war [126]. The Pan American Health Organization’s (PAHO) most recent estimate of depression prevalence in Guatemala was 3.7% in 2015 [127]. To our knowledge, no study has investigated depression among Indigenous people in the western highland region. Thirty-two percent of the study population screened for depressive symptoms using the CES-D questionnaire, which is higher than what other studies have reported for Guatemala; however, estimates for the prevalence of depression in Low- and Middle-Income Countries (LMICs) are as high as 24% in some areas [128].

The rates in this region may be higher than in other parts of the country due to circumstances specific to this area. Residents in these communities are of Indigenous Maya descent, and most came to live in the rural highlands to escape violence and discrimination because of their Indigenous status [89]. Access to economic opportunities, education, and healthcare is particularly limited in this area. The availability of land for cultivation in the highlands is diminishing rapidly, representing a decline in agricultural opportunities for young families [52,53]. A significant decrease in coffee prices and climate change-associated precipitation changes have made coffee production no longer sufficient for economic survival [38]. This area is also host to large-scale mining operations that displace Indigenous communities and threaten environmental health and agriculture without benefitting local residents [54,55]. Gang activity, including *Mara Salvatrucha (MS 13)* and *Barrio 18*, is established in San Marcos and making its way into rural communities, causing fear and disruption [51]. Securitization and militarization of migration have made migration more dangerous and expensive. Extortive activity and violent assault during the migrant journey and upon arrival at the border, as well as the threat of deportation once in the US, cause consistent stress and anxiety for loved ones remaining in the country of origin [56,57,58].

### 4.2. Aim 1: Emigrant Ties and Depression

Consistent with our hypotheses, having close ties with emigrants, and more of them, to the United States increased the likelihood of depressive symptoms within this rural migrant-sending community. We also found that women and those with lower education levels were more likely to experience depression. Only basic primary education is free in Guatemala, limiting access to many rural Indigenous residents. Male children are favored in continuing education because of traditional gender roles for women in the household, and much of this study population may be impacted by lower levels of education for Indigenous people during and after the civil war [129]. In our findings, individuals with more education were more likely to have contacts who emigrated to the United States, which may explain how their households had the economic resources to allow children to stop agricultural work and leave the home to attend school and pay for tuition beyond primary grades. Possible explanations for lower odds of depression among those with more education include the increased economic prospects it affords and opportunities to join existing contacts in the United States.

Having at least one close friend or neighbor who has emigrated was associated with depression. In the case of these rural communities, an explanation may be the pressure to provide what one’s neighbors are providing for their families in terms of quality of home construction or affording tuition for children. There may be some measure of self-worth through comparisons with an emigrant friend or neighbor [130,131]. Another possible explanation is that in these tightly bound communities, the supportive ties of friendship are more impactful at mitigating mental health distress than those provided by the nuclear family [132].

### 4.3. Aim 2: Social Network Characteristics as “Buffers” to Depression

As emigration from the region has not slowed and mental health resources are scarce, it is important to investigate possible assets that can be leveraged within communities to ease the burden felt by those remaining in the place of origin. Previous research has shown that a single close relationship may be enough to impact mental health [92,133]. Additionally, previous research reports varying mental health impacts of certain relationship types over others in a network [60,65,134,135,136,137]. In terms of protective factors against experiencing depression, a more connected network offered some protection against depression in individuals with emigrant ties. It is reasonable that an individual surrounded by people who are also close to one another experiences a certain degree of support and inclusivity. Having one’s mother or sibling in the home network also lowered the odds of depression, highlighting the importance of certain relationships in buffering the impacts of loss. Transnational family structures are navigated as a normalized part of life for these communities, but having direct, physical access to specific significant people like a mother and brothers or sisters offers some protection against poor mental health outcomes [60,65,92,133,134,138]. In terms of solutions for those living in this community, this suggests the opportunity for programs that increase connection between individuals outside of their own households. While relationships with mothers and siblings cannot be replaced or recreated, what they provide in terms of specific kinds of social support may be strengthened through network cohesion.

Curiously, individuals who named more people as part of their network (out-degree) were more likely to experience depression. This finding is contrary to some previous research, which found that more socially isolated people have a higher risk of depression and have reported the positive effect of network size against poor mental health outcomes [65,139,140]. There is evidence in the literature that more socially integrated people experience better mental health [92,141,142]. Studies have reported mental health benefits associated with larger networks and have used network size as a measure of support that lowers the odds of depressive symptoms [92]. However, there is also evidence in the literature that the quality of social support in relationships is more important for mental health than simply the existence of interpersonal relationships [143].

Further investigation of the relationship between the out-degree and depression yielded significance for an interaction with the out-degree. Stratification into subgroups by the out-degree suggested that those with higher numbers of emigrant ties are still more likely to be depressed than their peers with fewer or none, but the **odds of depression are lower for those in the high out-degree subgroup,** even with increasing numbers of emigrant ties. This may indicate that those with more emigrant ties still have higher odds of experiencing depression, but individuals with bigger social networks at home may reduce their odds of depression.

### 4.4. Gendered Differences in Network “Buffering”

To better understand why the out-degree is associated with depression, we explored what out-degree nominations looked like for individuals. Men seem to be driving this statistic, as those with higher proportions of nominees from within their households had increased odds of depression. And men with more “friends” outside of their household lowered their odds of depression. This relates to another finding that men with close friends or neighbors who emigrated had higher odds of depression. In these circumstances, men may benefit from more social support from outside of the responsibilities of the home, while for women, having some key relationships within the household was most significant. Though the literature about the importance of male friendships on the mental health of men in Latin America is scarce, there is evidence in research on Latinx populations for the essential support offered by non-familial relationships, especially for aging adults [144]. This finding offers an opportunity for further research on the role of non-household relationships on men’s mental health in the context of Indigenous, migrant-sending communities.

Analyses stratified by reported gender revealed some other differences in the buffering impact of social network characteristics for men and women. For men, having a spouse at home was associated with reduced odds of depression. It is reasonable that having spousal support in the home would be supportive of good mental health, and this is consistent with the literature [136,145]. Having their father in the home network was associated with depression for men. One possible explanation may be related to the pressure of “failed masculinity.” Research from Pérez (2014) argues that males experience a “sense of self” through migration. In this instance, thwarted masculinity could simply be a failure to provide, but a failure to realize aspirations of migration as a means to provide should also be considered as a version of that thwarted masculinity. When providing financially through migration is unrealized, it challenges masculinity and can create a perceived devaluation of self [48]. For men, these findings suggest an opportunity for programs strengthening male relationships and cooperative efforts outside of the household. Network ties that do not require responsibility of care for men and that foster understanding and mentorship, preventing feelings of failed masculinity, may provide some protection from depressive symptoms.

Consistent with mental health research worldwide, women in the study population experienced more depressive symptoms [146,147,148]. Although women are increasingly emigrating to the United States, they are still more likely to be left at home in Guatemala to care for children and older adults. For women, having either parent at home was associated with decreased odds of depression, which highlights the importance of those significant close relationships. The total degree, or all the people she nominated and who nominated her, was associated with depression for women. More social connections may actually increase stressors on women with limited resources, as they signal increased responsibility to provide social support to others [149]. Higher transitivity scores, or a more connected network, were a significant moderator in the relationship between emigrant ties and depression for women, suggesting that the effect of having emigrant ties on the probability of depression is buffered by this third variable of social network transitivity. There is evidence that transitivity in social networks is important for emotional support and that network cohesion is associated with better mental health outcomes [150,151]. In this community, it may be the case that the possible negative emotional impacts of having close ties with emigrants are mitigated for women with more cohesive support networks. However, the relationship between transitivity and network cohesion is nuanced. Social processes have been found to both increase and decrease depressive symptoms in highly transitive groups [152]. Homophily, or the tendency for people’s networks to be homogenous in socio-demographic, behavioral, and other characteristics, has been associated with transitivity in social networks [153].

Transitivity in networks has been linked to depression under this principle of homophily, suggesting that depressed individuals may form connections with other people also experiencing depression [65,154]. Highly transitive networks also do not allow for a major transgression from norms, and there is evidence of diffusion of depression in highly connected networks [155,156,157,158,159]. The varied nature of findings around transitivity, mental health, and social support indicates that further research is necessary to understand how transitive networks in this community provide relief from depressive symptoms for those with emigrant ties. Our findings suggest that a person in a more cohesive network fares better with social loss; thus, future research should test measures of homophily and explore whether an individual’s named alters also have someone who has emigrated. For women, these findings suggest an opportunity for programs supporting increased connectivity in feminine networks. Again, parental relationships that buffer against depression cannot be recreated; however, network connectedness through transitivity seems to be protective in this community and may offer areas for female community building and support. This must be approached cautiously, as larger networks without connectivity can result in increased social burden on women and the possibility of spreading depressive symptoms among stressed networks.

The culture of migration is experienced differently for men and women, and even for younger and older individuals. This can be understood as the responsibility of transnational cultural elaboration of care [41]. Young people may endure parental absence and fulfill educational responsibilities [52,160]. Older adults contribute by providing stable childcare, and women contribute to the household in the global chain of care by sacrificing physical proximity to their spouse and older children. Men also participate in the global care chain and often face challenges to their roles as husbands, fathers, and providers due to their extended and sometimes permanent absence or a failure to successfully migrate [161].

### 4.5. Study Limitations

This study has limitations. These data are from a non-random survey census of a purposively sampled village; therefore, findings are not generalizable to all people living in rural areas of Guatemala. The survey was a cross-sectional network study of a single village; therefore, it does not allow for an assessment of causality in the findings. There is also the possibility of social desirability bias when answering both migration and depression questions, as they were of a sensitive nature, which could have resulted in under-reporting of depressive symptoms and undocumented migration of friends and family. We only attempted to measure depressive symptoms as the main outcome within the scope of this study. Outcomes other than depression, including anxiety and the independent quantification of culturally relevant concepts like *nervios* and *tristeza*, should ideally also be explored. Within the scope of this study, the depressive symptom outcome was used as a starting point to explore how standardized and familiar measures may be used to quickly understand how migration impacts mental health. This survey was understandably somewhat difficult and burdensome for participants, as it brings up difficult and painful memories and topics. A more comprehensive and in-depth study should be the next step, including using diagnostic interviews with mental health professionals to validate the use of these instruments within these vulnerable populations. This outcome also fails to disentangle the nuanced intersection of trauma and resilience experienced within this population, including childhood adversity in its changing forms within each generation. The CES-D depression screening tool, while widely studied and validated, is not as sensitive as interview methods with mental health professionals in the detection of depressive symptoms. The CES-D tool has also not been validated in this specific population against interview methods with mental health professionals. Furthermore, there is no reliable mental health data reporting in Guatemala, and we did not have baseline depression scores for people living in the region of this study population, which limited our ability to interpret depression incidence found in this study; however, adding to the knowledge about mental health in this region with our findings on depression in this community is part of the value of this study.

## 5. Conclusions

The high number of out-migrants from rural communities in Central America is drastically altering traditional family and community structures. There are few allocated resources for mental health for Indigenous Maya living in rural locations. Mental health resources are centered in major metropolitan areas, which are virtually inaccessible for Indigenous people living in rural regions. This study begins to fill the gap in the literature on the mental health challenges of rural Indigenous populations living in communities that are strongly impacted by out-migration. Using socio-centric network data to map possible assets within communities, including how to leverage social networks, may provide opportunities for easing burdens on these populations.

Future research should focus on in-depth diagnostic interviews with mental health professionals to gain a more accurate characterization of depression and anxiety within this population. Not only do they face the challenges of high-volume migration, but their history of deep trauma and exposure to new forms of violence through gang activity and structural inequity creates an intersectionality for mental health impacts that are impossible to disentangle with standardized scales. Cultural concepts like *nervios* and *tristeza* should also be explored independently as measures of mental health distress within these populations.

In terms of social networks, future research should investigate network relationships and characteristics that exacerbate or improve poor mental health outcomes. These ties and characteristics are possible assets to be leveraged in mitigating the social costs of losing network ties to out-migration. A better understanding of the relationship between measures of degree and depression is also important. In this study population, it was unclear why, contrary to the existing literature, higher measures of degree were associated with depression, and why the impact of emigrant ties on depressive symptoms varied by levels of the out-degree. Also, the link between alcoholism in the household and depression, and the relationship to higher rates of alcoholism in households with emigrant ties, should be explored. The complexity of these relationships could not be addressed within the scope of this manuscript. The nature of depression within and apart from transitive groups should be further investigated, as previous studies have found that transitivity can guard against depression and exacerbate it in highly transitive groups. Strengthening social ties and transitivity in personal networks may be a tool to mitigate depressive symptoms among women remaining at home in this region. Finally, the link between emigrant ties and depression can be better understood by studying the temporal relationship between the number of ties, types of relationships lost, and the onset of depressive symptoms.

## Figures and Tables

**Table 1:** Summary statistics individual survey (*N* = 653).

Mean (SD) Age in years 33.96 (16.51)			
Mean (SD) CES-D depression score 12.38 (9.90)			
Total network density 0.00855			
	**Proportion of Total Sample**	**Proportion with CESD Score Above 16** **(*p-Value*)**	**Proportion with Emigrant Tie** **(*p-Value*)**
**CES-D score above 16**			
No	67%	-	52% (0.093)
Yes	33%	-	59%
**Emigrant in the United States**			
No	46%	29% (0.093)	-
Yes	54%	36%	-
**Sex**			
Female	59%	45% (<0.001)	53% (0.166)
Male	41%	16%	57%
**Marital status**			
Single	36%	19% (0.838)	55% (1.00)
Married/civil union	64%	33%	54%
**Education**			
None	39%	46% (<0.001)	50% (0.003)
Primary school	53%	23%	54%
More than primary	8%	30%	76%
**Religious affiliation**			
No	42%	28% (0.44)	52% (0.307)
Yes	58%	36%	56%
**Income sufficiency**			
Sufficient	27%	29% (0.313)	61% (0.055)
Not sufficient	73%	34%	52%
**Self-alcohol dependency**			
No	84%	31% (0.18)	53% (0.39)
Yes	16%	39%	58%
**Household alcohol dependency**			
No	63%	29% (0.016)	55% (0.727)
Yes	37%	39%	53%

**Table 2:** Bivariate regression on predictors of depressive symptoms (*N* = 653).

	Beta	SE	*p*
**Emigrant tie**			
No	Ref		
Yes	0.298	0.169	0.078
Number of emigrant ties	0.096	0.031	0.002
Age in years	0.013	0.005	<0.001
**Age categories**			
15–20	Ref		
21–29	0.486	0.247	0.049
30–43	0.927	0.238	<0.001
44–88	0.738	0.242	0.002
**Sex**			
Men	Ref		
Women	1.468	0.197	<0.001
**Marital status**			
Single	Ref		
Married/civil union	0.051	0.175	0.77
**Education**			
None	Ref		
Primary school	−1.082	0.18	<0.001
More than primary	−0.724	0.323	0.025
**Religious affiliation**			
No	Ref		
Yes	0.361	0.172	0.036
**Income sufficiency**			
Sufficient	Ref		
Not sufficient	0.211	0.191	0.27
**Household alcohol dependency**			
No	Ref		
Yes	0.425	0.171	0.013
**Friend/neighbor emigrated**			
No	Ref		
Yes	0.604	0.192	0.002
**Spouse emigrated**			
No	Ref		
Yes	0.426	0.374	0.25
**Parent emigrated**			
No	Ref		
Yes	−0.329	0.482	0.49
**Child emigrated**			
No	Ref		
Yes	−0.066	0.272	0.81
**Sibling emigrated**			
No	Ref		
Yes	0.211	0.198	0.29
**Spouse in home network**			
No	Ref		
Yes	−0.255	0.175	0.15
**Mother in home network**			
No	Ref		
Yes	−0.375	0.176	0.033
**Father in home network**			
No	Ref		
Yes	−0.273	0.175	0.12
**Sibling in home network**			
No	Ref		
Yes	−0.383	0.192	0.047
Total degree	0.033	0.012	0.006
In-degree	0.019	0.02	0.345
Out-degree	0.067	0.02	<0.001
Transitivity	−2.184	0.879	0.013

**Table 3:** Results of GLM logistic regression on predictors of depressive symptoms with binary yes/no for emigrant tie (*N* = 653).

	Model 1			Model 2			Model 3		
	Emigrant Tie Main Effect Adjusted for Total Degree			Emigrant Tie Main Effect with Transitivity Adjusted for Total Degree			Emigrant Tie Main Effect with Out-Degree		
	Beta	SE	*p*	Beta	SE	*p*	Beta	SE	*p*
**Emigrant tie**									
No	Ref			Ref			Ref		
Yes	0.496	0.188	0.008	0.474	0.189	0.012	0.489	0.189	0.01
Transitivity				−1.891	1.001	0.05			
Out-degree							0.044	0.021	0.039
Age in years	0.01	0.006	0.117	0.01	0.006	0.107	0.012	0.006	0.05
**Sex**									
Men	Ref			Ref			Ref		
Women	1.42	0.214	<0.001	1.401	0.215	<0.001	1.398	0.215	<0.001
**Marital status**									
Single	Ref			Ref			Ref		
Married/civil union	−0.126	0.206	0.541	−0.144	0.208	0.489	−0.112	0.205	0.585
**Education**									
None	Ref			Ref			Ref		
Primary school	−0.78	0.21	<0.001	−0.763	0.211	<0.001	0.737	0.213	<0.001
More than primary	−0.23	0.369	0.532	−0.171	0.371	0.644	−0.187	0.371	0.615
**Religious affiliation**									
No	Ref			Ref			Ref		
Yes	0.179	0.195	0.36	0.147	0.197	0.454	0.166	0.196	0.398
**Household alcohol dependency**									
No	Ref			Ref			Ref		
Yes	0.517	0.191	0.008	0.49	0.192	0.011	0.522	0.191	0.006
**Income sufficiency**									
Sufficient	Ref			Ref			Ref		
Not sufficient	0.348	0.211	0.099	0.331	0.212	0.118	0.368	0.212	0.082
Total degree	0.02	0.014	0.145	0.019	0.014	0.166			

**Table 4:** Results of GLM logistic regression on predictors of depressive symptoms with number of emigrant ties (*N* = 653).

	Model 1			Model 2			Model 3		
	Number Emigrant Ties Main Effect Adjusted for Total Degree			Number Emigrant Ties Main Effect with Transitivity Adjusted for Total Degree			Number Emigrant Ties Main Effect with Out-Degree		
	Beta	SE	*p*	Beta	SE	*p*	Beta	SE	*p*
Number Emigrant ties	0.1	0.035	0.004	0.097	0.035	0.005	0.099	0.035	0.004
Transitivity				−1.802	1.003	0.072			
Out-degree							0.039	0.021	0.066
Age in years	0.01	0.006	0.11	0.011	0.006	0.097	0.012	0.006	0.055
**Sex**									
Men	Ref			Ref			Ref		
Women	1.392	0.214	<0.001	1.374	0.216	<0.001	1.371	0.215	<0.001
**Marital status**									
Single	Ref			Ref			Ref		
Married/civil union	−0.102	0.207	0.622	−0.12	0.209	0.565	−0.094	0.206	0.649
**Education**									
None	Ref			Ref			Ref		
Primary school	−0.758	0.21	<0.001	−0.745	0.211	<0.001	−0.714	0.213	<0.001
More than primary	−0.222	0.373	0.551	−0.164	0.375	0.661	−0.173	0.375	0.644
**Religious affiliation**									
No	Ref			Ref			Ref		
Yes	0.156	0.196	0.428	0.126	0.197	0.524	0.14	0.197	0.476
**Household alcohol dependency**									
No	Ref			Ref			Ref		
Yes	0.481	0.192	0.012	0.456	0.193	0.018	0.487	0.192	0.011
**Income sufficiency**									
Sufficient	Ref			Ref			Ref		
Not sufficient	0.428	0.217	0.048	0.41	0.218	0.059	0.45	0.218	0.039
Total degree	0.015	0.014	0.272	0.015	0.014	0.296			

**Table 5:** Bivariate regression on predictors of depressive symptoms by sex (male, *n* = 269).

	Beta	SE	*p*
**Emigrant tie**			
No	Ref		
Yes	0.851	0.375	0.023
Number of emigrant ties	0.098	0.058	0.089
Age in years	−0.005	0.01	0.62
**Marital status**			
Single	Ref		
Married/civil union	0.407	0.214	0.057
**Education**			
None	Ref		
Primary school	−1.014	0.219	<0.001
More than primary	−0.253	0.452	0.576
**Religious affiliation**			
No	Ref		
Yes	0.026	0.217	0.904
**Income sufficiency**			
Sufficient	Ref		
Not sufficient	0.54	0.441	0.221
**Household alcohol dependency**			
No	Ref		
Yes	0.446	0.212	0.035
**Friend/neighbor emigrated**			
No	Ref		
Yes	0.486	0.241	0.044
**Spouse emigrated**			
No	Ref		
Yes	0.067	0.397	0.866
**Parent emigrated**			
No	Ref		
Yes	0.173	0.614	0.956
**Child emigrated**			
No	Ref		
Yes	0.173	0.346	0.617
**Sibling emigrated**			
No	Ref		
Yes	0.182	0.249	0.734
**Spouse in home network**			
No	Ref		
Yes	−0.862	0.34	0.011
**Mother in home network**			
No	Ref		
Yes	0.313	0.349	0.37
**Father in home network**			
No	Ref		
Yes	0.72	0.353	0.042
**Sibling in home network**			
No	Ref		
Yes	−0.076	0.396	0.849
Total degree	−0.009	0.027	0.736
In-degree	−0.127	0.109	0.244
Out-degree	0.052	0.023	0.027
Transitivity	−0.805	1.448	0.588

**Table 6:** Bivariate regression on predictors of depressive symptoms by sex (female, *n* = 384).

	Beta	SE	*p*
**Emigrant tie**			
No	Ref		
Yes	0.27	0.206	0.19
Number of emigrant ties	0.093	0.039	0.017
Age in years	0.03	0.007	<0.001
**Marital status**			
Single	Ref		
Married/civil union	0.051	0.175	0.77
**Education**			
None	Ref		
Primary school	−1.082	0.18	<0.001
More than primary	−0.724	0.323	0.025
**Religious affiliation**			
No	Ref		
Yes	0.361	0.172	0.036
**Income sufficiency**			
Sufficient	Ref		
Not sufficient	0.264	0.228	0.254
**Household alcohol dependency**			
No	Ref		
Yes	0.425	0.171	0.013
**Friend/neighbor emigrated**			
No	Ref		
Yes	0.604	0.192	0.002
**Spouse emigrated**			
No	Ref		
Yes	0.426	0.374	0.25
**Parent emigrated**			
No	Ref		
Yes	−0.329	0.482	0.49
**Child emigrated**			
No	Ref		
Yes	−0.066	0.272	0.81
**Sibling emigrated**			
No	Ref		
Yes	0.211	0.198	0.29
**Spouse in home network**			
No	Ref		
Yes	0.275	0.206	0.181
**Mother in home network**			
No	Ref		
Yes	−0.566	0.208	0.006
**Father in home network**			
No	Ref		
Yes	−0.523	0.209	0.012
**Sibling in home network**			
No	Ref		
Yes	−0.319	0.222	0.152
Total degree	0.042	0.015	0.006
In-degree	0.065	0.028	0.022
Out-degree	0.112	0.102	0.273
Transitivity	−2.698	1.123	0.016

**Table 7:** Results of GLM logistic regression on predictors of depressive symptoms by sex (female, *n* = 384).

	Model 1			Model 2			Model 3		
	Number Emigrant Ties Main Effect Adjusted for Total Degree			Number Emigrant Ties Main Effect with Transitivity			Number Emigrant Ties Main Effect with Out-Degree		
	Beta	SE	*p*	Beta	SE	*p*	Beta	SE	*p*
Number Emigrant ties	0.107	0.043	0.013	0.105	0.043	0.015	0.106	0.043	0.013
Age in years	0.02	0.008	0.014	0.023	0.008	0.006	0.023	0.008	0.005
**Marital status**									
Single	Ref			Ref			Ref		
Married/civil union	0.114	0.239	0.635	0.149	0.241	0.536	0.137	0.239	0.567
**Education**									
None	Ref			Ref			Ref		
Primary school	−0.662	0.256	0.01	−0.718	0.254	0.005	−0.619	0.259	0.017
More than primary	0.15	0.496	0.762	0.269	0.509	0.598	0.183	0.499	0.714
**Religious affiliation**									
No	Ref			Ref			Ref		
Yes	0.043	0.238	0.855	0.018	0.239	0.939	0.02	0.239	0.932
**Household alcohol dependency**									
No	Ref			Ref			Ref		
Yes	0.521	0.231	0.024	0.448	0.233	0.055	0.525	0.232	0.023
**Income sufficiency**									
Sufficient	Ref			Ref			Ref		
Not sufficient	0.37	0.253	0.146	0.309	0.255	0.225	0.386	0.254	0.129
Transitivity				−2.735	1.274	0.032			
Out-degree							0.051	0.025	0.04
Total degree	0.028	0.016	0.09						

## Data Availability

Due to the sensitive nature of this study topic (unauthorized migration and mental health) and potential harm to participants, data from this study cannot be made publicly available. For data inquiries, please contact the first author.

## Abbreviations

The following abbreviations are used in this manuscript:

CES-D	Center for Epidemiologic Studies-Depression scale
CEH	Comisión para el Esclarecimiento Histórico
GLM	Generalized Linear Regression
HCHS/SOL	Hispanic Community Health Survey/Study of Latinos
